# MTQA: Text-Based Multitype Question and Answer Reading Comprehension Model

**DOI:** 10.1155/2021/8810366

**Published:** 2021-02-18

**Authors:** Deguang Chen, Ziping Ma, Lin Wei, Jinlin Ma, Yanbin Zhu

**Affiliations:** ^1^School of Computer Science and Engineering, North Minzu University, Yinchuan 750021, China; ^2^School of Mathematics and Information Science, North Minzu University, Yinchuan 750021, China

## Abstract

Text-based multitype question answering is one of the research hotspots in the field of reading comprehension models. Multitype reading comprehension models have the characteristics of shorter time to propose, complex components of relevant corpus, and greater difficulty in model construction. There are relatively few research works in this field. Therefore, it is urgent to improve the model performance. In this paper, a text-based multitype question and answer reading comprehension model (MTQA) is proposed. The model is based on a multilayer transformer encoding and decoding structure. In the decoding structure, the headers of the answer type prediction decoding, fragment decoding, arithmetic decoding, counting decoding, and negation are added for the characteristics of multiple types of corpora. Meanwhile, high-performance ELECTRA checkpoints are employed, and secondary pretraining based on these checkpoints and an absolute loss function are designed to improve the model performance. The experimental results show that the performance of the proposed model on the DROP and QUOREF corpora is better than the best results of the current existing models, which proves that the proposed MTQA model has high feature extraction and relatively strong generalization capabilities.

## 1. Introduction

With the further research in natural language processing (NLP), lots of advancements have been achieved in many tasks, including the sentiment analysis [1–5], machine translation [6–8], intelligent question answering [9–11], and so on. As an important component of NLP, the function of reading comprehension covers collection information, knowledge storage, logical reasoning, and even a well-integrated subjective answering strategy. Therefore, the development of reading comprehension will give an enormous impetus in promoting the development of NLP and its commercial application. The accuracy of current deep learning models based on a single-mode reading comprehension corpus has reached or surpassed the average level of humans on corpora such as TriviaQA [12], SQuAD [13, 14], SearchQA [15], NarrativeQA [16], and CNN/DailMail [17] corpora. In order to promote the continuous development and progress in this field, researchers are devoted to proposing new corpora with wider coverage and more difficult questions and answers to meet research and development needs of researchers. In 2019, Dua [18] et al. completed a new corpus called QUOREF, which contains 10% multisegment corpus and expands the complexity of the corpus to a certain extent compared with single-segment question and answer. In the meantime, Dua et al. proposed the discrete reasoning over the content of paragraph corpus (DROP) [19], which is more difficult and complex to obtain accurate answer and has a wider range of fragment types such as numeric, date, and segment types (single-fragment and multifragment). Hence, it is more challenging to test and evaluate the performance of algorithms on DROP.

The corpus of DROP was proposed in order to promote the development in the NLP field of reading comprehension. However, a limited number of related and published articles implement experiments on the DROP corpus. Accordingly, a few related models have been presented in this field. The well-known models include NAQANet [19], NumNet [20], MTMSN [21], TbMS [22], and GENBERT [23]. However, in terms of their current performance, the F1 value of these models' performance is all about 80, which is much lower than that of the human level. Therefore, further research on this corpus is very challenging and necessary.

In this paper, a multitype question and answer reading comprehension model (MTQA) is proposed based on previous work on the DROP corpus. In general, in this paper, multilayer transformer is employed as the basic encoding and decoding structure. In addition, drawing on the multihead mechanism in MTMSN, the answer type prediction, segment, arithmetic operation, counter, and negation headers are added to the decoder. In order to improve the efficiency of cluster search algorithm in MTMSN, an absolute loss function is proposed in our model. Moreover, we modified the multilayer transformer model to adapt to the latest ELECTRA (efficiently learning an encoder that classifies token replacements accurately) model checkpoint. At the same time, inspired by GENBERT and Gururangan [24], part of the pseudo-corpus is generated and combined with the existing corpora for the secondary pretraining of the model. In general, the contributions of this paper can be listed as follows:

1. An innovative model (MTQA) is proposed. At the same time, the MTQA model is convenient and efficient in tasks from pretraining to downstream, which effectively combines strong supervised learning and weakly supervised learning. As a result, the performance of the MTQA model has been significantly improved than that of single-supervised model. It exceeds the highest performance of the existing model by 4.5+% on the DROP corpus.
2. This paper transferred the idea of generative adversarial networks (GANs), which have been used in the image field, into the MTQA model. The proposed MTQA model draws on the idea of GAN, applies GAN to natural language processing, and shares the ELECTRA checkpoints, which can avoid large-scale pretraining of the corpus.
3. This paper evaluated that MTAQ model has higher accuracy. Moreover, experimental results on the QUOREF corpus show that our model also achieved the highest accuracy, which proves that MTQA has a relatively strong generalization ability.

## 2. Related Work

The main work of this paper is implemented on the DROP corpus. According to different classification standards, the models involved in the DROP corpus can be divided into different categories. From the perspective of the label design, due to the complexity of the DROP corpus, the processed labels are not unique, so most models adopted weakly supervised learning methods. On the other hand, from the perspective of the model design, the current stage is mainly divided into two categories: arithmetic operation on external methods and arithmetic operations on the model itself, which is entrusting the arithmetic operations involved in the DROP corpus to external methods, and inferring the arithmetic operations based on the model's own capabilities. In the following sections, we describe these two methods.

### 2.1. Arithmetic Operations on External Methods

NAQANet is the first model designed for the DROP corpus. It includes four decoding heads to process the corpus: the paragraph fragment header, question fragment header, counting header, and arithmetic operation header. Among them, fragment headers (paragraph fragments and question fragments) are used to process single and multiple fragments in the DROP corpus, as well as part of the date type question and answer. On the other hand, the count and arithmetic operation headers are used to process the arithmetic operations in the DROP corpus and the arithmetic operations involving question and answer in the data type. In the NABERT + model, the encoding method in NAQANet is mainly substituted for the BERT method. At the same time, standard numbers and template operation are added to the arithmetic operation head, thus further improving the accuracy of the model. MTMSN has made the following improvements based on NABERT+. Firstly, an answer type header is added to predict the type of the answer. Secondly, under the condition of counting a large number of corpora, it is found that many situations are negative, so a negative header is added to deal with numerical calculation problems. Thirdly, a cluster search algorithm is added to the model to process the answers to the fragments. Through the three aforementioned improvements, the model performance has been greatly improved. TbMS improves the limited effect of multisegment processing in the previous model and has achieved better results on multisegment corpus, with an overall effect similar to that of the MTMSN. Similar model structures include TASE [26] model, etc.

### 2.2. Arithmetic Operations on the Model Itself

Yu et al. used models such as QANet + ELMo [27] and BERT [28] to train DROP corpus. However, the effect of their models was not very satisfactory. Currently, the GENBERT model is the first and only model that uses the internal parameters of the model to train on DROP corpus with ideal results. In addition, in this model, BERT's codec structure is employed to process the DROP corpus, while a segment decoding header is added to the decoder structure to process the segment corpus that appears in DROP. However, experimental results verified that the accuracy of such a model on DROP corpus is much lower. Therefore, in order to improve the accuracy, the model redesigned the pretraining checkpoint of BERT for a secondary pretraining. Firstly, a large number of pseudo-natural language corpora are generated for digital and text computing, and then BERT's masked language model (MLM) mechanism is combined to obtain its checkpoints for the secondary pretraining. Secondly, the DROP corpus is sent to the model with the secondary checkpoints for the downstream fitting training. The experimental results show that the performance of the model is equivalent to that of the MTMSN base downstream task. The main innovations and optimal results of the models are shown in Table 1.

The arithmetic operation of DROP is externally performed, which reduces the internal pressure of the model and improves its accuracy to a certain extent. However, the internal parameters of the model may result in overfitting, i.e., the model itself is not sufficiently trained and not convenient for model expansion. The arithmetic operation of DROP relies on the model itself to calculate and is not good at dealing with numerical reasoning problems, which leads to a much low performance of the model, while the model is more flexible. In this paper, we propose the MTQA model to achieve better generalization ability and higher performance.

## 3. MTQA Model

### 3.1. Problem Description

**Figure 1:**
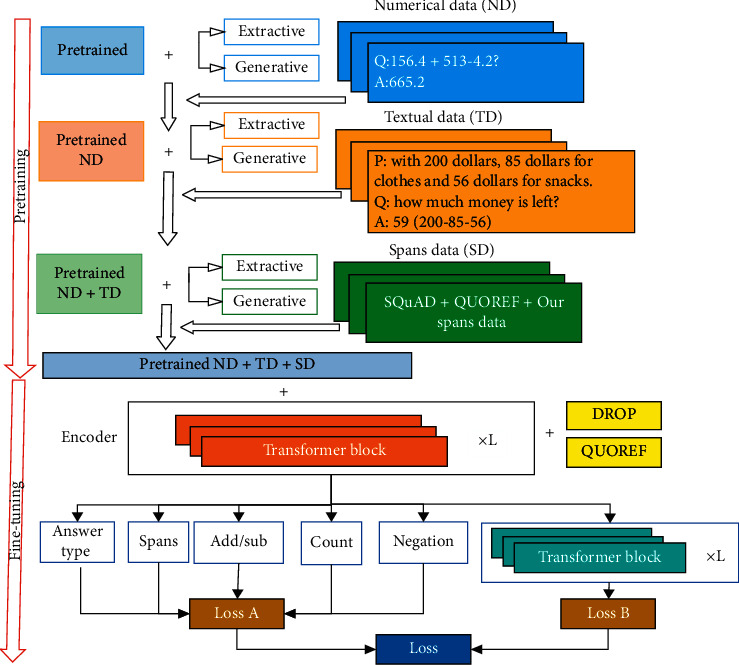
The flowchart of MTQA model.

Given a question-and-answer pair of text *X*=(*x*^*P*^, *x*^*Q*^) (corresponding words in *x*^*P*^ and *x*^*Q*^ are marked as *x*_*i*_^*P*^ and *x*_*j*_^*Q*^ respectively, where *i*, *j* are the subscripts of the corresponding order words), reason about the text based on the proposed question and get the answer *x*^*A*^, i.e., *p*max(*x*^*A*^*|*(*x*^*P*^, *x*^*Q*^)). The inference process involves adding and subtracting the numbers in the text, finding the maximum and minimum values, counting, selecting, and comparing. The flowchart of our model proposed in this paper is shown in Figure 1.

Firstly, we pretrain the model of MTQA. In the pretraining process, initial pretraining and secondary pretraining are involved. The pretrained results are saved as checkpoints for downstream tasks. Secondly, in the downstream tasks, the corpus and corresponding pretraining checkpoints are added to the model for fitting training, and thus the final results are obtained.

### 3.2. Pretraining

#### 3.2.1. First Pretraining

**Figure 2:**
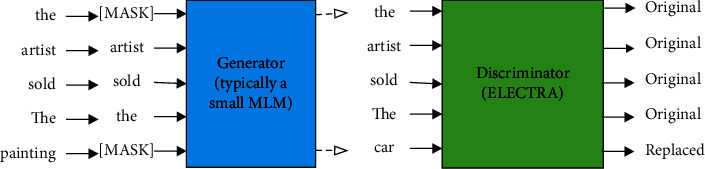
ELECTRA generator-discriminator.

ELECTRA has designed a pretraining mechanism, which is structured like a GAN, as shown in Figure 2. Under the condition of relatively fewer parameters and corpora, the performance of ELECTRA surpasses that of BERT. Generally speaking, the main contribution of ELECTRA is to propose a new pretraining mechanism, i.e., to change the BERT's MLM to a discriminative replacement detection mechanism (replaced token detection, RTD).

Specifically, an MLM generator is used to transform the input sentence, by covering some words, and then throw them to the discriminator, which can determine whether the words have been changed. In order to overcome the problem of gradient fragmentation caused by words discretization, the generator and discriminator are simultaneously trained. But the discriminator gradient is not passed to the generator. Furthermore, their joint loss function is minimized, and the formula is as follows:

(1) $$\begin{array}{c} \underset{\theta G,\theta D}{\min}\sum_{x\in \chi }\varsigma_{\text{MLM}}\left(x,\theta G\right)+\lambda \varsigma_{\text{Disc}}\left(x,\theta D\right), \end{array}$$

where *ς*_MLM_ is the loss function of the generator, *ς*_Disc_ indicates the loss function of the discriminator, *θ* means the optimization objective, and *χ* represents all text corpora. Since the task of the discriminator is relatively easy, the RTD loss is much smaller than that of MLM; hence, a larger coefficient *λ* is added to the discriminator. The pretraining checkpoint in this paper originates from ELECTRA since the ELECTRA model has certain advantages over the BERT pretraining model. More methods related to ELECTRA can be found in [29].

#### 3.2.2. Secondary Pretraining

Due to a wide range of sources of pretraining corpus of the ELECTRA, it has generality but not pertinence. It was inspired by the conclusion, proposed by Gururangan [24], that we can get a better effect if we can get more task-related data and continue pretraining. In addition, ELECTRA pretraining relatively lacks for the numerical reasoning and continuous word fragments of the DROP corpus, which can affect the model performance in the downstream fitting training to a certain extent. At the same time, the checkpoint of the ELECTRA pretraining model is slightly smaller than that of BERT under the same circumstances. Hence, in order to achieve a fair comparison and a higher accuracy of our model, we propose to carry out the secondary pretraining for the number type and the continuous word segment type in the DROP corpus.

We mainly utilize a new mixture corpus of program-generated pseudo-natural language corpus and a small amount of corpora produced artificially for the secondary pretraining as a result of the limited human and financial resources. The process of the secondary pretraining is as follows.

Firstly, we design programs to create a numerical corpus (numerical data, ND), produced by 6 standard templates, and a textual corpus (textual data, TD), produced by numbers (NUM), entities (ENT), containers (CONT), and attributes (ATTR). The ND and TD corpora are used to solve the numerical reasoning problems. For a detailed production process of ND and TD, refer to [23].

Secondly, in order to overcome the lack of a continuous word segment corpus (spans data), we make a new corpus by adopting the existing SQuAD, QUOREF, and part of multisegment corpus made by ourselves manually. This new corpus is mainly to extract the task of questions and answering. Then, features extracted from the three types of corpora are made into a suitable form for the model and saved as pkl files format for subsequent reading. Finally, the corresponding pkl files are sent into the network for secondary pretraining (the network structure is described later). In view of the characteristics of our model, we mainly use weakly supervised learning to improve correctness of decision making to the most extent.

Furthermore, three types of corpora are trained simultaneously, and three sets values of EM and F1 will be produced in this process. These three sets of F1 values are averaged. If the average value is greater than the current best F1 value (the initial F1 value is 0), the checkpoint is saved; otherwise, the current checkpoint is discarded and we proceed to the next round of pretraining.

### 3.3. Model Structure

#### 3.3.1. Basic Codec Structure

In the upstream task, ELECTRA is employed, so the encoding structure of our model adopts the corresponding structure based on ELECTRA which is essentially an improvement on the pretraining model of BERT even if their encoding structures are very similar.

*(1) Basic Encoding Structure*. Firstly, the words in each question and answer corpus are segmented according to the vocabulary and divided into corresponding subwords. Secondly, the words are embedded (subwords, positions, and labels are correspondingly added), and then the processed sentences are sent to the model's multilayer transformer encoder for feature extraction (assuming that there is an L-layer transformer). Finally, the last four features (HL − 3, HL − 2, HL − 1, HL) of the Transformer encoding are retained, and after transformation, they are recorded as (FI0, FI1, FI2, FI3) to prepare for decoding.

*(2) Basic Encoding and Decoding Structure*. Based on the transformer encoder, an L-layer transformer decoder is added to construct the basic decoding structure of the model.

**Figure 3:**
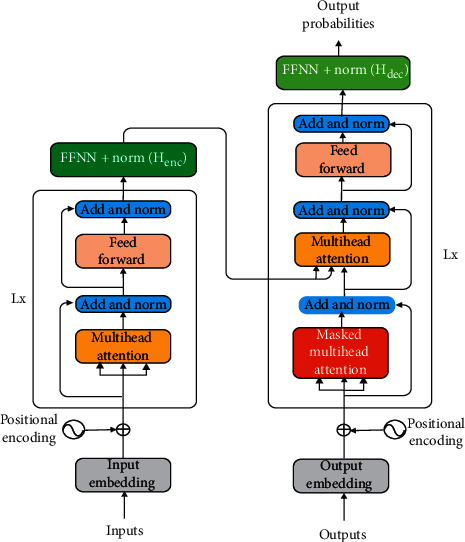
Basic encoding and decoding structure of multilayer transformer.

In order to make the weight of ELECTRA, which is used to initialize the encoder, applicable for decode, we combine the weights of the encoder and decoder so that the weight of ELECTRA is completely used to initialize the model. Meanwhile, we add an *H*_enc_ layer above the encoding layer to transform the context semantics, such that they can learn different representations. The basic encoder and decoder structure is shown in Figure 3, and the formula form is as follows:

(2) $$\begin{array}{c} \text{FI}=\text{layer}-\text{norm}\left(\text{gule}\left(W\cdot H_{\text{enc}}\right)\right), \end{array}$$

where *W* is the weight matrix, gule denotes the activation function, and layer-norm is the layer regularization operation. In the same way, we use the same method to process the decoded answer *H*_dec_ and obtain the decoded answer FO; the formula is as follows:

(3) $$\begin{array}{c} \text{FO}=\text{layer}-\text{norm}\left(\text{gule}\left(W\cdot H_{\text{dec}}\right)\right). \end{array}$$

#### 3.3.2. Additional Decoding Structure

Our model refers to MTMSN. One head is the answer type prediction header, used to predict the type of the answer (addition and subtraction, text segment, question segment, count or negative). The second one is the segment header, used to process extractive answer segments. The remaining three heads are used to process numeric operations: an arithmetic head, a counting head, and a negation head. The five-head mechanism is briefly introduced as follows.

**Figure 4:**
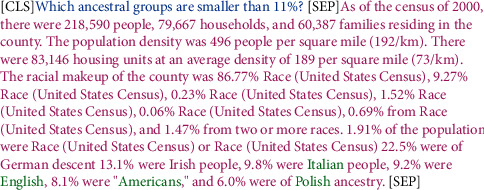
Feature segmentation form.

*(1) Answer Type Prediction Head*. For the sake of predicting the output type of the answer, FI_2_ is divided into the question feature *Q*_2_ and text segment feature *P*_2_ at the corresponding position where [SEP] first appears; the form is shown in Figure 4. Then, the problem feature vector *h*^*Q*2^ and the text segment feature vector *h*^*P*2^ are calculated as follows:

(4) $$\begin{array}{c} \alpha^{Q}=\text{soft max}\left(W^{Q}Q_{2}\right),\;h^{Q2}=\alpha^{Q}Q_{2},\\ \alpha^{P}=\text{soft max}\left(W^{P}P_{2}\right),\;h^{P2}=\alpha^{P}P_{2}, \end{array}$$

where *W*^*Q*^ and *W*^*P*^ are parameter matrices. By using *h*^*Q*2^ and *h*^*P*2^, different prediction types can be calculated by the following formula:

(5) $$\begin{array}{c} P^{\text{type}}=\text{soft max}\left(\text{FFN}\left(\left[h^{Q2};h^{P2};h^{\text{CLS}}\right]\right)\right), \end{array}$$

where *h*^CLS^ is the vector corresponding to CLS in FI_2_, which contains the semantic information of the entire question and answer pair, FFN represents a feedforward neural network with two-layer linear mapping using the GELU activation function, and *P*^type^ is the type of the predicted answer.

*(2) Segment Prediction Head*. To extract the answer segment from the text corpus or question corpus, it is necessary to find the start and end position marks of the answer in the input “[CLS] question sequence [SEP] text sequence [SEP].” In order to reflect the difference between the start position and the end position, different vector representations are used, respectively, and the corresponding formulas are as follows:

(6) $$\begin{array}{c} \beta^{Q2}=\text{soft max}\left(\text{FFN}\left(Q2\right)\right),\;v^{Q2}=\beta^{Q2}Q2, \end{array}$$

where *v*^*Q*0^, *v*^*Q*1^, and *v*^*Q*2^ are similar to the calculation method. After that, we can calculate the start and end vectors in the problem; the corresponding formula is

(7) $$\begin{array}{c} {\bar{\text{FI}}}^{\text{start}}=\left[{\text{FI}}_{2};{\text{FI}}_{0};v^{Q2}⊗{\text{FI}}_{2};v^{Q0}⊗{\text{FI}}_{0}\right],P^{\text{start}}=\text{soft max}\left(W^{S}{\bar{\text{FI}}}^{\text{start}}\right),\\ {\bar{\text{FI}}}^{\text{end}}=\left[{\text{FI}}_{2};{\text{FI}}_{1};v^{Q2}⊗{\text{FI}}_{2};v^{Q1}⊗{\text{FI}}_{1}\right]\left(\right]P^{\text{end}}=\text{soft max}\left(W^{E}{\bar{\text{FI}}}^{\text{end}}\right), \end{array}$$

where ⊗ represents the outer product of the vectors *v* and FI. The vectors *P*^start^ and *P*^end^ are the probability of each subword as the starting position and ending position in the question, respectively. In the same way, the calculation in the text is performed.

*(3) Arithmetic Expression Header*. In order to perform arithmetic operations, all the numbers in the question and the text are extracted and recorded as *N*=(*n*_1_, *n*_2_,…, *n*_*M*_), where *M* is the largest subscript of the number. Each number may be added, subtracted, or set to zero. The probability that the *i*^th^ subscript is recorded as addition, subtraction, or zero can be calculated as follows:

(8) $$\begin{array}{c} p_{i}^{\text{sign}}=\text{soft max}\left(\text{FFN}\left[n_{i};h^{Q2};h^{P2};h^{\text{CLS}}\right]\right). \end{array}$$

The corresponding symbolization processing is performed on the corresponding position of the number of *P*_*i*_^sign^ (i.e., the corresponding position is added, subtracted, or set to zero), and the result of the *i*^th^ digital position can be obtained. Adding all the digital positions together can achieve the final result of the arithmetic expression.

*(4) Counting Head*. Considering the actual counting ability, the calculation range set in this paper is set from 0 to 9. Firstly, a standard vector is created by the following formula:

(9) $$\begin{array}{c} \alpha^{U}=\text{soft max}\left(W^{U}U\right),\;h^{U}=\alpha^{U}U, \end{array}$$

where *h*^*U*^ is the one-hot code from 0 to 9 and the number of counts *P*^count^ can be calculated as follows:

(10) $$\begin{array}{c} P^{\text{count}}=\text{soft max}\left(\text{FFN}\left(\left[h^{U};h^{Q2};h^{P2};h^{\text{CLS}}\right]\right)\right). \end{array}$$

*(5) Negation Head*. As one of the characteristics of the corpus, many data items appear in the negative form. Therefore, negation head is set. The negative form is to use 100 minus the current number to obtain the current position number. *N* in this header is the same as *N* in the arithmetic header, and its negation probability formula is as follows:

(11) $$\begin{array}{c} P_{i}^{\text{negation}}=\text{soft max}\left(\text{FFN}\left[n_{i};h^{Q2};h^{P2};h^{\text{CLS}}\right]\right). \end{array}$$

### 3.4. Loss Function

#### 3.4.1. Weak Supervision Loss Function

Six decoding headers are involved in this work, among which the answer type, segment prediction, arithmetic expression, counting, and negation headers are all trained in a weakly supervised way. Taking an arithmetic expression as an example, assuming there are *n* candidate answers as *x*={*x*_1_, *x*_2_,…, *x*_*n*_}, the logarithm of the softmax of the candidate answer *x* can be calculated according to following formula:

(12) $$\begin{array}{c} {\text{loss}}_{\text{Arithmetic}}=\sum_{i=1}^{n}\log\frac{\exp\left(x_{i}\right)}{\sum_{j=1}^{n}\exp\left(x_{j}\right)}, \end{array}$$

where loss_Arithmetic_ is the loss function of the arithmetic expression. In a similar way, the corresponding loss functions of the other four headers: loss_type_, loss_spans_, loss_Count_, and loss_nagetion_, can be obtained. Then, these five sets of loss functions can be added to obtain the weakly supervised loss function loss_weak_, as in the following formula:

(13) $$\begin{array}{c} {\text{loss}}_{\text{weak}}=\underset{t}{\sum }{\text{loss}}_{t},\;t\in \left\{\text{type},\text{spans},\text{count},\text{nagetion},\text{arithmetic}\right\}. \end{array}$$

Since these loss functions drop quickly, it is easy to cause the gradient to disappear and diffuse; we utilize the log-sum-exp function for processing, according to the following formulas:

(14) $$\begin{array}{c} \text{constant}=\underset{i}{\max}\left({\text{loss}}_{{\text{weak}}_{i}}\right),\\ {\text{loss}}_{1}=\text{constant}+\log\sum_{i}e^{{\text{loss}}_{{\text{weak}}_{i}}-\text{constant}}, \end{array}$$

where loss_weak_*i*__ is the loss rate of the *i*^th^ corpus in a batch. In this way, the weakly supervised loss function loss_1_ can be obtained.

#### 3.4.2. Basic Decoding Loss Function

The basic decoding head adopts strong supervised learning to adjust the internal parameters of the model. Since the answer types include numbers, the composition of the numbers is variable, which is impossible to be listed in the vocabulary. To obtain better fitting the answer, we segment the number type labels, for example: 12345 is divided into 1, ##2, ##3, ##4, ##5. Then, the loss rate is calculated according to the cross-entropy, and the corresponding formula is as follows:

(15) $$\begin{array}{c} {\text{loss}}_{2}=\frac{1}{n}\sum_{i}^{n}-t_{i}\log\left(p_{i}\right)-\left(1-t_{i}\right)\log\left(1-p_{i}\right), \end{array}$$

where *t*_*i*_ is the *i*^th^ label of the real answer labels and *p*_*i*_ is the *i*^th^ number of the predicted answer labels. The function of the basic decoding is to add a strong supervised learning mechanism to assist the weakly supervised learning.

### 3.5. Absolute Loss Function

Due to digital labels of the weakly supervised learning in the process, the labels themselves are partially incorrect, which results in incorrect training, thereby affecting the overall performance of the model. To overcome this problem, in MTMSN, a cluster search algorithm is used for auxiliary training and prediction [21]. More concretely, the most likely first three answers are selected as the correct answer during training. However, in recurring experiments, we found that the accuracy of the model during the training process cannot be effectively improved by the cluster search algorithm, since it still uses weakly supervised learning. For this reason, we add an absolute loss function in the training process of the cluster search. The added absolute loss function uses strong supervision learning, thus improving the performance of the model to a certain extent.

Specially, the steps are as follows. Firstly, a complete answer and answer type are extracted from the triples (i.e., text, question, answer) as the standard answer. Secondly, for answers with a number or date type, predictions are made after training. According to the characteristics of the model, the predicted answers form a vector group. Then, the cluster search algorithm is employed to search the most likely answer vector as the predicted answer. Finally, compared with the predicted answer and the answer type, respectively, the real answer and the answer type are defined and calculated by the formulas as follows:

(16) $$\begin{array}{c} {\text{loss}}_{\text{answertype}}=\left\{\begin{array}{cc} 0, & \text{if }P_{\text{answertype}}=T_{\text{answertype}},\\ a, & \text{if }P_{\text{answertype}}\neq T_{\text{answertype}}, \end{array}\right) \end{array}$$

(17) $$\begin{array}{c} {\text{loss}}_{\text{answer}}=\left\{\begin{array}{cc} 0, & \text{if }P_{\text{answer}}=T_{\text{answer}},\\ b, & \text{if }P_{\text{answer}}\neq T_{\text{answer}}, \end{array}\right) \end{array}$$

where *P* represents the predicted answer and *T* denotes the real one. Furthermore, loss_3_ calculated by this strong supervision method is

(18) $$\begin{array}{c} {\text{loss}}_{3}={\text{loss}}_{\text{answertype}}+{\text{loss}}_{\text{answer}}. \end{array}$$

At this point, we have all the loss functions: loss_1_loss_2_, and loss_3_, and the total loss function loss can be expressed as

(19) $$\begin{array}{c} \text{loss}={\text{loss}}_{1}+\lambda {\text{loss}}_{2}+\mu {\text{loss}}_{3} \end{array}$$

On account of a certain degree of difference between the loss function numbers calculated by different methods, the two parameters of *λ*, *μ* are set to balance and eliminate the difference.

## 4. Experiment

This section introduces the corresponding experimental design and results, aiming to prove that the proposed model of MTQA can achieve high accuracy and has a certain scalability.

### 4.1. Experimental Platform and Environment

All the experiments in this work are carried out on two platforms. One is a Win10 platform with 2 Intel (R) Xeon (R) Gold 6154 processors, 256GB memory, 2 Nvidia TITAN V graphics cards, Python version 3.6.8, and torch version 1.1.0. The system mainly performs base and related basic tasks. The other platform is a CentOS 7.5.1804 platform with Intel (R) Xeon (R) Gold 6140, 360GB memory, 2 Tesla V100 graphics cards, Python version 3.6.8, and torch version 1.4.0. The large and related basic tasks are mainly completed on this system.

### 4.2. Introduction to Corpora

The corpora used in this work are DROP and QUOREF. The DROP corpus is constructed by more than 96,000 question-answer (QA) pairs from more than 6,700 text fragments from Wikipedia. It is divided into three sets: training, development, and test. The training set contains about 77,000 QA pairs, and the development and test sets contain approximately 9,500 QA pairs each. Each text fragment contains about 200 words, and each question contains about 11 words. The corpus includes addition and subtraction, comparison, selection, counting, multiple fragments, etc. The QUOREF corpus constructs over 24,000 QA pairs from more than 4,700 text fragments from Wikipedia. It is divided into three sets: training, development, and test. The training set contains about 19,000 questions, and the development and test sets contain about 2,500 questions each. Each text fragment contains about 384 words, and each question contains about 17 words. This corpus contains single-segment and multisegment QAs, of which multisegment QAs account for about 10% of the total number of QAs.  Table 2 shows the relevant statistics of the two corpora.

### 4.3. Secondary Pretraining

This section describes the secondary pretraining performed on the ELECTRA checkpoint on the ND, TD, and SD corpora. The purpose of the secondary pretraining is to make the pretraining targeted so as to better fit the downstream tasks. We perform secondary pretraining at the base and large checkpoints of ELECTRA, respectively. Similar to the two trends, we choose large checkpoint to give explanation.

**Figure 5:**
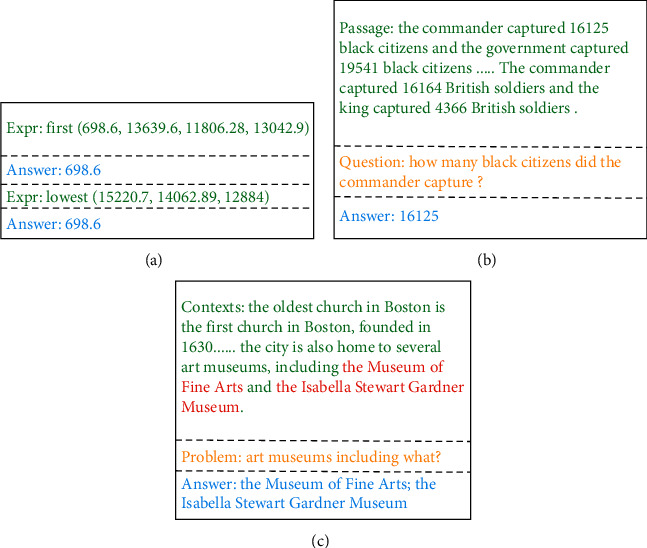
Sample corpora.

Firstly, we developed a large-scale corresponding corpus. For the ND corpus, 510,000 pieces of corpus (500,000 training and 10,000 test) are program-generated. For the TD corpus, 110,000 corresponding text fragments are program-generated, and each text fragment contains 3 to 8 QAs of the corpus, which produces about 550,000 training corpus pieces and 55,000 test corpus pieces. The SD corpus includes 330,000 (300,000 training and 30,000 test) from SQuAD, QUOREF, and our hand-made multisegment corpus.  Figure 5 shows the corpus styles of ND, TD, and SD, and Table 3 shows the situation of each corpus.

**Experiment 1 .**

Secondary pretraining. In order to verify the precision of secondary pretraining, secondary pretraining is performed based on the large checkpoints. The batch size of ND, TD, and SD is set to 12, and the learning rate is set to 2*e* − 6. The corresponding F1 and EM values are recorded from step 4000, once every 2000 steps. The F1 and EM values of the three corpora are shown in Figure 5.

**Figure 6:**
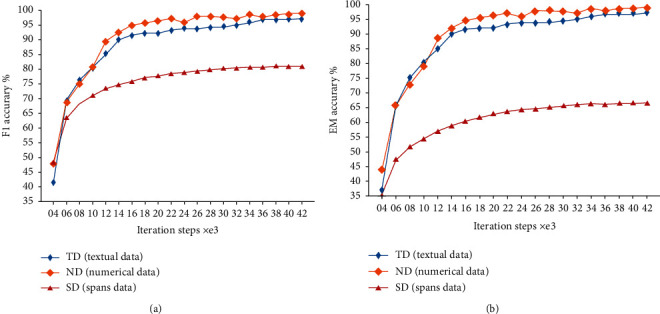
Secondary pretraining effect. (a) The F1 values of the three corpora. (b) The EM value of the three corpora.

From Figure 6, the following can be observed. (1) After the curves tend to be stable, the accuracies of ND and TD of the F1 and EM values in Figures 6(a) and 6(b) reach up to more than 97%, which indicates that the program-generated pseudo-corpus is relatively simple and the training is easier. (2) Compared with ND and TD in Figures 6(a) and 6(b), the accuracy of SD is relatively lower (F1 accuracy is only about 80% and EM value is only about 65%), demonstrating that the artificially constructed corpus has a certain degree of difficulty and is more challenging to be trained than the pseudo-corpus. (3) The difference of SD curves between F1 and EM is about 15%, whereas those of ND and TD are relatively smaller, which may be due to a large number of multisegment corpus pieces in SD, indicating that the training on the SD corpus is more difficult from a side view.

### 4.4. Model Building

This section mainly focuses on describing the key steps in the construction and validating the efficiency of the proposed model through horizontal and vertical comparison. In addition, we compare our model, i.e., MTQA, with the existing models to verify its effectiveness.

**Experiment 2 .**

Model building experiment. Firstly, we use the ELECTRA checkpoint to verify its validity by replacing BERT pretraining checkpoint of the MTMSN with the ELECTRA checkpoint, modifying the corresponding code to make it effective and recording it as MTMSN_Electra_. Then, we carry out the corresponding experiments on the base and large checkpoints. Secondly, we add the five decoding heads of MTMSN to the basic decoding structure of multilayer transformer. Meanwhile, the absolute loss function is added. And the model is marked as MTQA, which performs related experiments on the model at the BERT and ELECTRA checkpoints, respectively, marking as MTQA_Bert_ and MTQA_Electra_, respectively. Thirdly, the ELECTRA checkpoint is pretrained for the second time, and then the secondary pretraining checkpoint is put into MTQA for corresponding training and test, i.e., recorded as MTQA_pre_Electra_. Each model is run 10 batches, and the corresponding results are shown in Table 4.

The following conclusions can be drawn from Table 4. (1) On the pretraining model, the batch size and learning rate fluctuate to a certain extent because we choose batch quantity and learning rate with the highest precision, so setting them within the appropriate range will have a certain impact on the accuracy. (2) Throughout the first and second rows in base or large, the ELECTRA checkpoint is indeed more effective than the BERT checkpoint. (3) The results at the first and third rows in base or large show that the MTQA model is more effective than the MTMSN model. (4) The results at the second, third, and fourth rows in base or large verify that the ELECTRA checkpoint and MTQA represent a mutually reinforcing process. (5) The results at the fourth and fifth rows of base or large demonstrate that purposefully secondary pretraining can effectively improve the performance of the model, thereby improving the recognition performance accuracy (since the corpora used in the secondary pretraining is relatively small, we imagine that with the corpora increasing, it is very likely that the performance of the model can continue to improve). (6) Under the same model conditions of base and large, adding a new module will increase the accuracy. Especially the accuracy of base is significantly greater than that of large, which is due to the low relative accuracy of the base corpus. Moreover, when the new module is added, the feature extraction ability is correspondingly strengthened.

After the model is successfully constructed, we further validate by comparing our model with other existing models horizontally. The results are shown in Table 5.

In Table 5, it is demonstrated clearly that our model (MTQA) can achieve higher accuracy than the compared models based on either base or large, while MTMSN and TbMS can achieve higher accuracy. On the base pretraining models, our model exceeds the MTMSN model EM value by 7.50 percentage points and the F1 value by 7.18 percentage points. On the large pretraining models, our model exceeds the TbMS model EM value by 4.34 percentage points and the MTMSN model F1 value by 4.72 percentage points. In short, compared with MTMSN, TbMS, and other models, our model has obvious advantages, which proves its effectiveness, whereas there is still a certain gap compared with human level.

### 4.5. Decoding Head Performance and Model Generalization Capabilities

In this section, we design the third decoding head performance experiment, i.e., in each subexperiment, we remove a decoding head to observe the performance of the model, aiming to explore how much influence each decoding method has on the performance of our model, which can make it convenient for subsequent optimization of our model.

From Experiment 2 above, it can be seen that accuracy variation degree of pre_Electra_Base is greater than pre_Electra_Large. Although different secondary pretraining models will have different accuracies, owing to decoding head comparative experiments, they will have the same trends of the accuracy changes. In consideration of computing power, related decoding head comparative experiments are conducted only on pre_Electra_Base.

**Experiment 3 .**

Decoding head comparative experiments are conducted when the batch size is set to 8, the learning rate is 3*e* − 5, and 10 batches of training are performed. The experimental results are shown in Table 6.

The following aspects can be observed from Table 6. (1) The five types of decoding headers (segment, arithmetic operation, counting, negation, and basic decoding headers) have a positive influence on the model. (2) The fragment and arithmetic operation headers have greater impact degree on our model. The possible reasons could be the following two points: these headers are more capable of extraction features compared with other headers and their corpora occupy a larger proportion in the DROP corpus. (3) The poor performance of the basic decoding head in this experiment may be caused by the negative impact of the other four heads and the absolute loss function of the model. (4) The learning rate is in accordance with the batch size, which may result in a slight unfairness to the contrast head, because the learning rate of each head may be slightly different.

Generally, a multihead mechanism may result in the generalization ability of the model being not strong enough. For this purpose, we select the QUOREF corpus to conduct related experiments to verify strong generalization ability of our model. QUOREF corpus has stronger diversity, which can be more difficult to be trained compared with SQuAD and can be evaluated by stable performance metric of our model.

**Experiment 4 .**

QUOREF experiment. Since the SD corpus in the secondary pretraining contains SQuAD, QUOREF, and other corpora, it is unfair and reasonable to choose the pre_Electra_Base or pre_Electra_Large pretraining model only for the QUOREF corpus experiment. Thus, we only directly use MTQA_Electra_Base_ and MTQA_Electra_Large_ without pretraining for the corresponding experiments. In practical terms, we set the fragment header and the basic decoding header to be true, the other headers are set to be false (because they are not used), the batch size is set to 10, and we perform the relevant experiments. The experimental results are shown in Table 7.

The following aspects can be noticed from Table 7. (1) BERT QA and XLNet QA are the base experiments of the QUOREF corpus. Their EM and F1 values are 64.52% and 71.49%, respectively. TASE_IO_ has the highest performance among existing works on the QUOREF corpus. (2) Our model achieved an EM value of 72.56 and an F1 value of 78.23 on the basis of Electra_base pretraining respectively, which indicates the performance of our model on the base exceeded that on the QUOREF corpus. (3) Our model achieved an EM value of 81.34% based on the Electra_Large pretraining and the F1 value of 86.84%, which surpasses the TASE_IO_'s EM value by 1.94% and F1 value by 1.94%, achieving the best value of the current QUOREF corpus. In a word, it is verified that our model has strong generalization ability and superior performance.

## 5. Conclusion

In this article, we proposed the model MTQA and evaluated it by performing corresponding experiments on multiple pretraining checkpoints of BERT and ELECTRA. Moreover, we mixed some pseudo-corpus and real corpus based on the ELECTRA checkpoint for secondary pretraining of our own checkpoint. The DROP and QUOREF corpora are used for the experiments. Compared with the existing models, the proposed model can achieve higher accuracy while also having stronger generalization ability. In addition, the accuracy of the DROP and QUOREF corpora is improved to a certain extent, which remains to be further improved in the text-based multitype question and answer reading comprehension model.

However, there is still a big gap between the performance of our model and the average level of human beings. In the next stage of work, we will proceed to merge the decoding head and improve the internal computer system of the transformer to reduce the amount of model parameters while improving the performance.

## Figures and Tables

**Table 1:** Optimal results and innovation of the models.

Model design perspective	Model	Results		Innovations
		*EM*	*F*1	
Operations on external methods	NAQANet	46.20	49.24	The first model built for DROP corpus, with four decoding heads
	NABERT+	64.61	67.35	Switch to BERT encoding based on NAQANet model
	MTMSN	76.68	80.54	Add two decoding heads and cluster search algorithm based on NABERT+
	TbMS	76.91	79.92	Improve and increase the answer prediction algorithm
	TASE	—	—	

Operations on the model itself	QANet + ELMo	27.71	30.33	Put the DROP corpus into the model for training and testing
	BERT_Base_	30.10	33.36	
	GENBERT	68.20	72.80	Use transformer internal structure for decoding and secondary pretraining

**Table 2:** Statistics of DROP and QUOREF corpora.

Corpora	Statistic	Training	Development	Test
DROP	Number of passages	5,565	582	588
	Avg. passage length	213	191	195
	Number of questions	77,409	9,536	9,622
	Avg. question length	11	11	11

QUOREF	Number of passages	3,771	454	477
	Avg. passage length	384	381	385
	Number of questions	19,399	2,418	2,537
	Avg. question length	17	17	17

**Table 3:** Pretraining corpora.

Corpus type	Corpus	Training set	Testing set
Generative	ND (numerical data)	500,000	10,000
	TD (textual data)	550,000	55,000
Manual construction	SD (spans data)	300,000	30,000

**Table 4:** Accuracy of key steps in model construction.

Pretrained model	Model	Accuracy		Epoch	Learning rate
		*EM*	*F*1		
Base	MTMSN_Bert_Base_ [21]	68.17	72.81	—	—
	MTMSN_Electra_Base_	71.49	75.95	8	3e − 5
	MTQA_Bert_Base_	69.59	74.87	13	5e − 5
	MTQA_Electra_Base_	74.62	78.98	8	3e − 5
	MTQA_pre_Electra_Base_	75.67	79.99	8	3e − 5

Large	MTMSN_Bert_Large_ [21]	76.68	80.54	—	—
	MTMSN_Electra_Large_	77.65	82.01	6	3e − 6
	MTQA_Bert_Large_	77.96	81.78	8	5e − 6
	MTQA_Electra_Large_	80.26	84.45	6	9e − 6
	MTQA_pre_Electra_Large_	81.25	85.26	12	5e − 6

**Table 5:** Comparison with existing models.

Models	Pretraining	Accuracy		Epoch	Learning rate
		*EM*	*F*1		
QANet + ELMo [27]	—	27.71	30.33	—	—
BERT [28]	Base	30.10	33.36	—	—
NAQANet [19]	—	46.20	49.24	—	—
MTMSN [21]	Base	68.17	72.81	—	—
	Large	76.68	80.54	—	—
TbMS [22]	Base	66.91	70.55	—	—
	Large_SQuAD	76.91	79.92	—	—
MTQA	pre_Electra_Base	75.67	79.99	8	3*e* − 5
	**pre_Electra_Large**	**81.25**	**85.26**	**12**	**5e −** **6**
Human performance [19]	—	92.38	95.98	—	—

**Table 6:** Decoding head comparative experiments.

Models	Accuracy	
	*EM*	*F*1
MTQA_pre_Electra_Base_	**75.67**	**79.99**
Remove segment prediction head	45.82	46.60
Remove arithmetic expression head	52.68	56.87
Remove counting head	69.69	73.69
Remove negation head	72.78	76.95
Remove basic decoding head	74.72	79.18

**Table 7:** QUOREF experiments.

Model	Accuracy		Epoch	Learning rate
	*EM*	*F*1		
QANet [30]	34.41	38.26	—	—
BERT QA [28]	58.44	64.95	—	—
XLNet QA [18]	64.52	71.49	—	—
CorefRoBERT_large_ [31]	74.94	81.71	—	—
TASE_IO_ [26]	79.4	84.9	—	—
MTQA_Electra_Base_	72.56	78.23	10	5*e* − 5
MTQA_Electra_Large_	**81.34**	**86.84**	**9**	**5e − 6**
Human performance [18]	86.75	93.41	—	—

## Data Availability

The data used to support the findings of this study have been deposited in https://s3-us-west-2.amazonaws.com/allennlp/datasets/drop/drop_dataset.zip and https://allennlp.org/quoref.

## References

[1] Xue W., Li T. Aspect based sentiment analysis with gated convolutional networks.

[2] Hu M., Liu B. Mining and summarizing customer reviews.

[3] Phan M. H., Ogunbona P. O. (2020). Modelling context and syntactical features for aspect-based sentiment analysis. *Proceedings of the 58th Annual Meeting of the Association for Computational Linguistics*.

[4] Gao Z., Feng A., Song X., Wu X. (2019). Target-dependent sentiment classification with BERT. *IEEE Access*.

[5] Liu Z., Lu C., Huang H., Lyu S., Tao Z. (2020). Hierarchical multi-granularity attention- based hybrid neural network for text classification. *IEEE Access*.

[6] Mathur N., Baldwin T., Cohn T. (2020). Tangled up in BLEU: reevaluating the evaluation of automatic machine translation evaluation metrics. *Proceedings of the 58th Annual Meeting of the Association for Computational Linguistics*.

[7] Ren S., Wu Y., Liu S., Zhou M., Ma S. (2020). A retrieve-and-rewrite initialization method for unsupervised machine translation. *Proceedings of the 58th Annual Meeting of the Association for Computational Linguistics*.

[8] Niu X., Mathur P., Dinu G., Onaizan Y. A. (2020). Evaluating robustness to input perturbations for neural machine translation. https://arxiv.org/abs/2005.00580.

[9] Saxena A., Tripathi A., Talukdar P. (2020). Improving multi-hop question answering over knowledge graphs using knowledge base embeddings. *Proceedings of the 58th Annual Meeting of the Association for Computational Linguistics*.

[10] Zheng B., Wen H., Liang Y. (2020). Document modeling with graph attention networks for multi-grained machine reading comprehension. https://arxiv.org/abs/2005.05806.

[11] Jayaratne M., Jayatilleke B. (2020). Predicting personality using answers to open-ended interview questions. *IEEE Access*.

[12] Joshi M., Choi E., Weld D. S., Zettlemoyer L. (2017). TriviaQA: a large scale distantly supervised challenge dataset for reading comprehension,” in association for computational linguistics (acl) vancouver, Canada. https://arxiv.org/abs/1705.03551.

[13] Rajpurkar P., Zhang J., Lopyrev K., Liang P. SQuAD: 100,000+ questions for machine comprehension of text.

[14] Rajpurkar P., Jia R., Liang P. (2018). Know what you don’t know: unanswerable questions for SQuAD. *Proceedings of the 56th Annual Meeting of the Association for Computational Linguistics*.

[15] Dunn M., Sagun L., Higgins M. (2017). A new Q&A dataset augmented with context from a search engine. https://arxiv.org/abs/1704.05179.

[16] Kočiský T., Schwarz J., Blunsom P. (2018). The NarrativeQA reading comprehension challenge. *Transactions of the Association for Computational Linguistics*.

[17] Hermann K. M., Kočiský T., Grefenstette E. (2015). Teaching machines to read and comprehend. https://arxiv.org/abs/1506.03340.

[18] Dua D., Wang Y., Dasigi P., Stanovsky G., Singh S., Gardner M. Quoref: a reading comprehension dataset with questions requiring coreferential reasoning.

[19] The Association for Computational Linguistics DROP: A reading comprehension benchmark requiring Discrete reasoning over paragraphs.

[20] Ran Q., Lin Y., Li P., Zhou J., Liu Z. (2019). NumNet: machine reading comprehension with numerical reasoning. https://arxiv.org/abs/1910.06701.

[21] Hu M., Peng Y., Huang Z., Li D. (2019). A multi-type multi-span network for reading comprehension that requires discrete reasoning. https://arxiv.org/abs/1908.05514.

[22] Efrat A., Segal E., Shoham M. (2019). Tag-based multi-span extraction in reading comprehension. https://arxiv.org/abs/1909.13375.

[23] Geva M., Gupta A., Berant J. (2020). Injecting numerical reasoning skills into language models. https://arxiv.org/abs/2004.04487.

[24] Gururangan S., Marasović A., Swayamdipta S. (2020). Don’t stop pretraining: adapt language models to domains and tasks. *Proceedings of the 58th Annual Meeting of the Association for Computational Linguistics*.

[25] Goodfellow I. J., Abadie J. P., Mirza M. Generative adversarial nets.

[26] Segal E., Efrat A., Shoham M., Globerson A., Berant J. (2019). A simple and effective model for answering multi-span questions. https://arxiv.org/abs/1909.13375.

[27] Yu A. W., Dohan D., Luong M. T. Fast and accurate reading comprehension by combining self-attention and convolution.

[28] Devlin J., Chang M. W., Lee K., Toutanova K. (2018). BERT: pre-training of deep bidirectional transformers for language understanding. https://arxiv.org/abs/1810.04805.

[29] Clark K., Luong M. T., Le Q. V., Manning C. D. ELECTRA: Pre-training text encoders as discriminators rather than generators.

[30] Yu A. W., Dohan D., Luong M. T. QANet: combining local convolution with global self-attention for reading comprehension.

[31] Ye D., Lin Y., Du J., Liu Z., Sun M., Liu Z. (2020). Coreferential reasoning learning for language representation. https://arxiv.org/abs/2004.%2006870.

